# Non-local interaction in discrete Ricci curvature-induced biological aggregation

**DOI:** 10.1098/rsos.240794

**Published:** 2024-09-04

**Authors:** Jyotiranjan Beuria, Laxmidhar Behera

**Affiliations:** ^1^ IKSMHA Center, IIT Mandi, Mandi, India; ^2^ IKS Research Center, ISS Delhi, Delhi, India; ^3^ Department of Electrical Engineering, IIT Kanpur, Kanpur, India

**Keywords:** biocomplexity, biophysics, applied mathematics

## Abstract

We investigate the collective dynamics of multi-agent systems in two- and three-dimensional environments generated by minimizing discrete Ricci curvature with local and non-local interaction neighbourhoods. We find that even a single effective topological neighbour suffices for significant order in a system with non-local topological interactions. We also explore topological information flow patterns and clustering dynamics using Hodge spectral entropy and mean Forman–Ricci curvature.

## 1. Introduction

Biological aggregation is one of the most ubiquitous and spectacular displays of coordinated behaviour of a collection of self-propelled particles [1–3]. The universality of this phenomenon can be seen in systems of very different sizes and scales, such as the flocking of birds [4,5], schooling of fish [6–8], bacterial colonies [9,10], locust swarms [11], sheep herds [12,13], and even human crowding [14–16] or robot swarming [17]. One of the characteristics of such a coordinated motion is the emergence of order in the system through the exchange of information among the agents. Several models for collective motion have been developed, primarily through local microscopic interaction mechanisms. Also, some recent studies have shown that active agents can undergo flocking without even explicitly aligning with neighbours [18–20]. However, a common feature of biological aggregation is to spread the consensus direction of motion among the constituent agents.

The study of collective motion is an active area of research both theoretically [18] and experimentally [21]. One of the most popular models of biological aggregation is the Vicsek model [22]. In this model, during each time step, the velocity of each agent is adjusted to match the average velocity of its neighbouring agents. Additionally, a random unit vector, scaled by a fixed noise strength, is added to this adjusted velocity. All the agents update their positions simultaneously at every time step based on the orientations. The interaction radius r defines the neighbourhood of interaction. Any two neighbouring agents must be separated by less than or equal to r. In other words, agents can only be influenced by their neighbours up to a radial distance r. In this model, an interesting phenomenon happens that all agents’ headings will converge to the same value or consensus for high group densities and low noise levels. The collective motion reaches a consensus faster with a larger interaction radius r, implying a denser interaction network and faster information flow.

Most of the models for collective motion are primarily based on local or short-range interaction neighbourhoods. Interestingly, some recent studies have examined interaction mechanisms extending beyond strict locality [23–26]. Proper consideration of non-local interactions is essential when the sensory systems of animals or birds are known to support such interactions physiologically. Natural swarms often exhibit long-ranged correlations [27], and many flocking animals or birds rely heavily on their vision [28,29], which can extend well beyond the immediate flock size. A study by Strandburg-Peshkin *et al*. [30] has demonstrated that biological organisms efficiently determine their movement directions using line-of-sight interactions. Notably, models with non-local interactions can easily produce velocity correlations across large distances [26]. This condition is more stringent for models when interactions are only local.

On top of that, the information flow among the agents is best modelled using a topological neighbourhood. The research conducted by Ballerini *et al*. [4] on starling flocks revealed that interaction among individuals is topological. Each agent coordinates with a fixed number, approximately seven, of its closest neighbours, regardless of their distances. This finding contradicts the assumptions made by most models of self-organized collective motion, which typically rely on metric interactions. Also, purely metric interactions require a much larger number of interacting neighbours [31,32]. Ballerini *et al*. have argued that topological interactions provide stronger cohesion than metric interactions, making them more effective from an anti-predatory perspective. Also, comparing metric to topological models in three dimensions, topological models exhibit superior stability [31], especially with spatially balanced neighbour selection, supporting findings from starling flock experiments [4]. This spatially balanced topological model requires fewer interacting neighbours. One of the goals of this work is to demonstrate the efficacy of discrete Ricci curvature-driven topological interaction in reducing the number of neighbours to as low as one.

From the above discussions, we realize that non-locality and the number of interacting neighbours are crucial for biological aggregation. Thus, we present a topological model that leverages the metric-based features and non-locality by constructing the Vietoris–Rips complex. In this model, an agent’s new orientation is not obtained by averaging over all possible nearest metric or topological neighbours. Instead, we prescribe a mechanism for following the direction of the most curved nearest neighbour(s) within an annular region around the agent. This model represents the entire system at a particular time step as a topological space using a Vietoris–Rips complex. The Ricci curvature measures how much the volume of a small ball in space changes compared to Euclidean space. We calculate a variant of discrete Ricci curvature by Forman [33] for the edges and vertices in this simplicial complex.

From a geometric perspective, the Forman–Ricci curvature (FRC) measures the information flow at the ends of edges within a network [34]. A large negative value for FRC of an edge indicates a higher spread of information at the ends of an edge. Such an edge has numerous neighbouring edges at both vertices, resembling a funnel connecting many other vertices. Interestingly, it has been observed that most real-world networks possess negative FRCs [35] for the edges. Thus, we expect that minimizing FRC while selecting interacting neighbours will favour biological aggregation. We also study the Hodge spectral entropy [36,37] that might offer insights into the higher-order information flow in the topological space formed by the agents. However, before we describe the model and the numerical simulations, we delve into a brief mathematical introduction to the rich field of topology and discrete Ricci curvature.

## 2. Forman–Ricci curvature and topology

### 2.1. Simplicial complex of the point cloud

**Figure 1.**
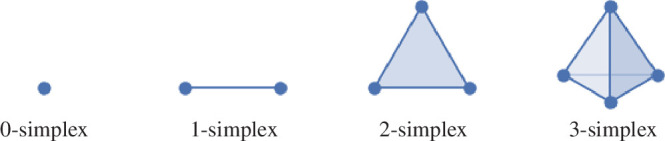
0-simplex is a vertex, 1-simplex is an edge, 2-simplex is a filled triangle and 3-simplex is a filled tetrahedron.

Constructing simplicial complexes is central to computational geometry techniques such as topological data analysis. It offers a systematic approach to constructing topological spaces using basic combinatorial units called simplices, facilitating the study of complex geometries through more manageable combinatorial and counting tasks. A k-dimensional simplex arises by taking a convex hull of k+1 points (see figure 1). For example, a 0-simplex corresponds to a vertex, a 1-simplex to an edge, a 2-simplex to a filled triangle and a 3-simplex to a filled tetrahedron. This construction can also be generalized to higher-dimensional polytopes. In a k-dimensional simplex, simplices with dimensions <k constitute its faces. Thus, for a 2-simplex, its edges and vertices constitute the faces.

Let $X=\{x_{0},x_{1},...,x^{m-1}\}\in {\mathbb{R}}^{m}$ represent a point cloud data, where each point $x_{i}$ resides in ${\mathbb{R}}^{n}$. Let r denote a fixed radius. The Vietoris–Rips complex of X is an abstract simplicial complex. Its k−simplices consist of (k + 1)-tuples of points in X such that the pairwise distance between them is less than or equal to r. This maximal radial limit r is also referred to as the filtration parameter. Mathematically, the Vietoris–Rips complex, also known as the Rips complex K, is expressed as:


(2.1) $$K=\{\sigma \subseteq X\;|\;d(x_{i},x_{j})\leq r\;\;\forall x_{i}\neq x_{j}\in \sigma \},$$

where $d(x_{i},x_{j})$ denotes the Euclidean metric between two points.

### 2.2. Constructing chain complexes

A k−chain, denoted by $C_{k}$, represents a formal sum of a subset of k−simplices belonging to the simplicial complex K. It is expressed as:


(2.2) $$C_{k}=\sum_{i}\alpha_{i}\sigma_{k}^{i}\;,$$

where $\sigma_{k}$ represents the k−simplex and $\alpha_{i}$ is a real number ($\alpha_{i}\in \mathbb{R}$). It is to be noted that $C_{k}$ forms an abelian group under component-wise addition. The k−chain group is generated by the k−cycles, which are k−chains without boundaries. The k−th boundary operator $\partial_{k}$ on a k−simplex $\sigma_{k}$ with vertices $\left(v_{0},v_{1},...,v_{k}\right)$ is defined as:


(2.3) $$\partial_{k}(\sigma_{k})=\sum_{i=0}^{k}(-1{)}^{i}(v_{0},v_{1},...,{\hat{v}}_{i},...,v_{k}),$$

where ${\hat{v}}_{i}$ denotes the vertex removed from $\sigma_{k}$. This operation maps a k−chain $C_{k}$ to a (k−1)-chain $C_{k-1}$. A chain complex is formed by the sequence of boundary operators acting on the chain groups:


(2.4) $$C_{k}\overset{\partial_{k}}{\to }C_{k-1}\overset{\partial_{k-1}}{\to }C_{k-2}\;\;...\;\;C_{1}\overset{\partial_{1}}{\to }C_{0}\overset{\partial_{0}}{\to }0.$$

The kernel of the boundary operator $\partial_{k}$ consists of all k-chains $C_{k}$ that have no boundary, while the image of $\partial_{k}$ is the set of (k−1)-chains $C_{k-1}$ that represent the boundaries of k-chains $C_{k}$. This can be mathematically expressed as


(2.5) $$\;\;\;\;\;\;\;\;\;\;\;ker(\partial_{k})=\{c\in C_{k}\;|\;\partial_{k}C_{k}=0\}\;im(\partial_{k})=\{d\in C_{k-1}\;|\;\exists c\in C_{k}:d=\partial_{k}(c)\}.$$

We note that k-boundaries are elements of $\text{im}(\partial_{k+1})$, whereas elements of $\text{ker}(\partial_{k})$ correspond to k-cycles. Moreover, under addition, the sets of k-boundaries $B_{k}$ and k-cycles $Z_{k}$ form abelian subgroups of $C_{k}$. Since k-boundaries are also k-cycles, it is important to observe that $\text{ker}(\partial_{k})\subset \text{im}(\partial_{k+1})$. With $B_{k}\subset Z_{k}\subset C_{k}$, the groups $B_{k}$, $Z_{k}$ and $C_{k}$ exhibit a nested structure. The quotient group that represents cycles modulo boundaries is called the k-th homology group, or $H_{k}$. It can be stated as:


(2.6) $$H_{k}=\frac{Z_{k}}{B_{k}}=\frac{\text{ker}(\partial_{k})}{\text{im}(\partial_{k+1})}.$$

Here, $H_{k}(K)$ represents the quotient vector space whose generators are given in terms of k-cycles that are not boundaries of any (k+1)-simplices. The rank of $H_{k}(K)$ is often referred to as the k-th Betti number $\beta_{k}(K)$. The Betti number $\beta_{k}(K)$ indicates the number of k-dimensional holes in the simplicial complex K that are not boundaries of any (k+1)-simplices. For example, $\beta_{0}(K)$ is the number of connected components in the simplicial complex. Euler characteristic χ is a topological invariant of the topological space. It is expressed as an alternate sum of Betti numbers $\beta_{k}$. Mathematically,


(2.7) $$\chi =\sum_{k=0}^{n}(-1{)}^{k}\beta_{k}.$$

### 2.3. Forman Ricci curvature

Let α and $\bar{\alpha }$ denote k-dimensional simplices within the simplicial complex K. If there exists a simplex β in K such that both β>α and $\beta >\bar{\alpha }$, then α and $\bar{\alpha }$ possess a common co-face β, and they are termed as upper adjacent. Similarly, α and $\bar{\alpha }$ are considered lower adjacent if they share a common face γ, a (k−1)-simplex with γ<α and $\gamma <\bar{\alpha }$. When α and $\bar{\alpha }$ are either lower or upper adjacent but not both, they are referred to as parallel. The FRC is expressed as [33]


(2.8) $$R_{k}(\alpha )=N(\text{Upper adjacent simplices})\;\;\;\;\;\;\;\;\;\;\;\;\;\;\;+N(\text{Lower adjacent simplices})\;\;\;\;\;\;\;\;\;\;\;\;\;\;\;\;\;\;\;\;\;\;\;\;\;\;\;\;\;\;\;\;\;\;-N(\text{Parallel simplices}).$$

In the context of weighted simplicial complexes with weights denoted as w, $R_{k}(\alpha )$ is expressed as


(2.9) $$\begin{array}{l} \begin{array}{rl} R_{k}(\alpha )= & w_{\alpha }\left[\sum_{\beta >\alpha }\frac{w_{\beta }}{w_{\alpha }}+\sum_{\gamma <\alpha }\frac{w_{\gamma }}{w_{\alpha }}\right]\\ \;- & w_{\alpha }\sum_{\bar{\alpha }\neq \alpha }\left[\sum_{\beta >\alpha ,\bar{\alpha }}\frac{\sqrt{w_{\alpha }w_{\bar{\alpha }}}}{w_{\beta }}-\sum_{\gamma <\alpha ,\bar{\alpha }}\frac{w_{\gamma }}{\sqrt{w_{\alpha }w_{\bar{\alpha }}}}\right]. \end{array} \end{array}$$

Considering up to 1-simplex in simplicial complex K, FRC for an edge reduces to


(2.10) $$\begin{array}{l} \begin{array}{rl} R_{1}(\alpha )= & w_{\alpha }\left(\sum_{\gamma <\alpha }\frac{w_{\gamma }}{w_{\alpha }}-\sum_{\bar{\alpha }\neq \alpha }\sum_{\gamma <\alpha ,\bar{\alpha }}\frac{w_{\gamma }}{\sqrt{w_{\alpha }w_{\bar{\alpha }}}}\right) \end{array} \end{array}.$$

Recently, there has been considerable interest in using FRC to study real networks [35,38–41] we encounter in various fields.

### 2.4. Hodge spectral entropy

The Hodge Laplacian is computed with respect to boundary operators. The Hodge Laplacian $L_{\left[n\right]}$ on a d-dimensional simplicial complex is defined for each n=0,…,d as


(2.11) $$L_{\left[0\right]}=L_{\left[0\right]}^{up}\;\;\text{for}\;n=0,$$

(2.12) $$L_{\left[n\right]}=L_{\left[n\right]}^{up}+L_{\left[n\right]}^{down}\;\;\text{for}\;n>0,$$

where $L_{\left[n\right]}^{up}$ and $L_{\left[n\right]}^{down}$ encode the diffusion from n-simplices to n-simplices via (n+1)-simplices and (n−1)-simplices, respectively. They can be expressed in terms of the boundary operators as follows:


(2.13) $$L_{\left[n\right]}^{down}=B_{n}B_{n}^{†},$$

(2.14) $$L_{\left[n\right]}^{up}=B_{n+1}^{†}B_{n+1}.$$

Using the Hodge Laplacian, we compute the von Neumann entropy for the simplicial complex as follows [36]:


(2.15) $$S_{n}^{hs}=-\text{Tr}(\rho_{n}\;ln\;\;\rho_{n}),$$

where $\rho_{n}$ is expressed in terms of the Hodge Laplacian as follows:


(2.16) $$\rho_{n}=\frac{e^{-\beta L_{\left[n\right]}}}{\text{Tr}(e^{-\beta L_{\left[n\right]}})},$$

where β is the damping factor and is different from the Betti numbers $\beta_{k}$ discussed earlier.

From a computational point of view, $S_{n}^{hs}$ can also be expressed in terms of the partition function $Z_{n}$ and eigenvalues of $L_{\left[n\right]}$ as follows:


(2.17) $$S_{n}^{hs}=\beta \;\left\langle \lambda_{n}\right\rangle +\;ln(Z_{n}),$$

(2.18) $$Z_{n}=\text{Tr}(e^{-\beta L_{\left[n\right]}}),$$

(2.19) $$\left\langle \lambda_{n}\right\rangle =\frac{\sum_{i}e^{-\beta \lambda_{i}(L_{\left[n\right]})}\lambda_{i}(L_{\left[n\right]})}{Z_{n}},$$

where $\lambda_{i}(L_{\left[n\right]})$ is the ith generic eigenvalue of $L_{\left[n\right]}$. With these mathematical preliminaries introduced above, we are ready to explore the model based on discrete Ricci curvature.

## 3. Ricci curvature-based non-local model

The model consists of N self-propelled agents moving at a constant speed ($v_{0}$) in a periodic box of length L and dimension d. The orientation of the agents keeps changing randomly. The model we consider in this work is a variant of the standard Vicsek model [2,22]. The Vicsek model is simple yet very powerful in predicting collective behaviour. The velocity of an agent is expressed as follows:


(3.1) $${\vec{v}}_{i}(t+1)=\frac{1}{|N_{i}|}\sum_{j\in N_{i}}{\vec{v}}_{j}(t)+v_{0}\eta {\hat{r}}_{i}(t),$$

where

— ${\vec{v}}_{i}(t+1)$ is the velocity of agent i at time t+1.
— $|N_{i}|$ represents the number of nearest neighbours of agent i.
— ${\vec{v}}_{j}(t)$ is the velocity of neighbour j of agent i at time t.
— η is the noise strength.
— ${\hat{r}}_{i}(t)$ is a random unit vector representing noise.

This equation represents the update rule for each agent’s velocity in the Vicsek model, where agents adjust their velocities to align with the average velocity of their neighbours plus a random noise term. The variants of the Vicsek model differ in how an agent’s nearest neighbours are selected. There are two primary variations, one with a topological neighbourhood and the other with a metric neighbourhood.

**Figure 2.**
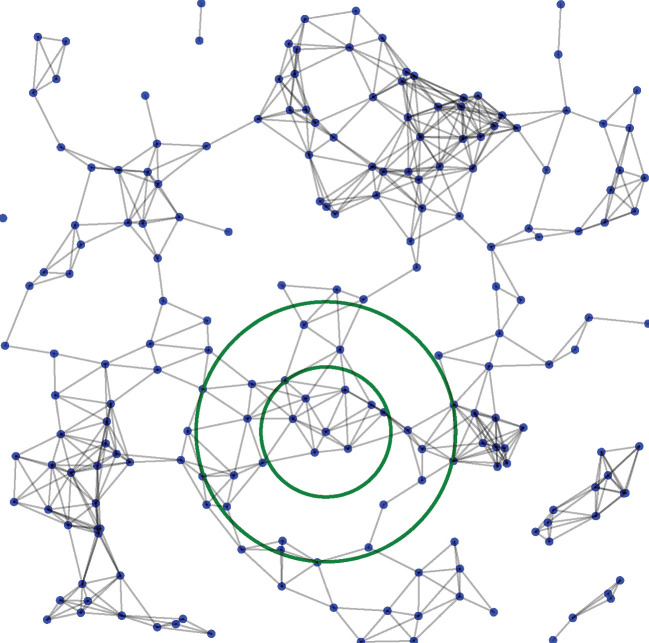
Schematic representation of the simplicial complex (up to edges) structure and the neighbouring agents in an annular region around an agent.

In figure 2, we suggest a non-local scheme for finding the most effective nearest neighbours. In the standard Vicsek model, the neighbours are found within a cutoff radius around the agent. Following King and Turner [26], we construct an annular disk of inner radius ($r_{min}$) and outer radius ($r_{max}$) around every agent. Traditional network models find the k−nearest neighbours of an agent and average their orientations to update the agent’s orientation up to a noise factor governed by η. However, we first chose to embed the entire system in a Vietoris–Rips complex. Thus, rather than dealing with fragmented portions of the system, we wish to capture both the local and non-local factors in deciding the new orientation of the agent.

The non-locality enters in two folds. First, we exclude the closest agents by choosing $r_{min}>0$. Second, instead of averaging over all the effective agents in the annular disk, we perform a weighted averaging of the orientations of the agents featuring the lowest FRC. We assign weights to the nodes and edges in the simplicial complex formed by the ensemble of agents. This process can also be extended to higher-order simplices for more precise results. Due to computational limitations, we restrict the homology dimension to 1. The weighting scheme is as follows.

Each edge’s weight, denoted as $w_{ij}$, is determined by the sum of the weights of the two vertices it connects, divided by $1+r_{ij}$, where $r_{ij}$ represents the distance between the vertices. For each edge connecting vertices i and j, the weight $w_{ij}$ is given by:


(3.2) $$w_{ij}=\frac{w_{i}+w_{j}}{1+r_{ij}},$$

where $w_{i}$ and $w_{j}$ are the weights of vertices i and j given by $w_{i}=1+\mathrm{degree}(i)$. This weight assignment scheme ensures that each edge’s weight is influenced by the combined weights of its connected vertices and inversely proportional to their distance. Also, the vertices with a larger number of connecting edges get larger weights.

The vertex FRC $R_{i}$ for agents is calculated as the sum of $R_{ij}$ over all edges incident to the ith vertex. Mathematically, it can be expressed as [42]:


(3.3) $$\begin{array}{ccc} & R_{i}=\sum_{j}R_{ij}, & \end{array}$$

where $R_{ij}$ represents the curvature associated with the edge connecting the ith vertex to its neighbouring vertices. This vertex curvature measure aggregates of the curvature contributions from all edges incident to a particular vertex, providing insights into the curvature at individual vertices within the network [34].

To determine the updated direction of movement of an agent, we select the k vertices with the lowest $R_{i}$ values, where $R_{i}$ represents the vertex curvature. Let $v_{i}$ denote the velocity vector associated with the i-th vertex. The weighted average velocity ${\vec{v}}_{i}(t+1)$ is then calculated as follows:


(3.4) $$\begin{array}{ccc} & {\vec{v}}_{i}(t+1)=v_{0}\frac{\sum_{i=1}^{k}w_{i}\cdot {\vec{v}}_{i}(t)}{\left\|\sum_{i=1}^{k}w_{i}\cdot {\vec{v}}_{i}(t)\right\|}+v_{0}\eta {\hat{r}}_{i}(t), & \end{array}$$

where $w_{i}$ represents the weight assigned to the ith vertex, and k is the number of effective interacting vertices. This procedure ensures that the collective direction of movement is influenced by the directions of the most curved agents from the chosen k agents. In case of only one effective nearest neighbour, the expression becomes


(3.5) $$\begin{array}{ccc} & {\vec{v}}_{i}(t+1)={\vec{v}}_{R_{min}^{i}}(t)+v_{0}\eta {\hat{r}}_{i}(t), & \end{array}$$

where ${\vec{v}}_{R_{min}^{i}}$ is the velocity of the most curved agent for i-th agent. As we will see in the numerical results, following the most curved agent can also bring appreciable order within the system. Thus, it is an economical model in terms of the number of nearest neighbours to be considered.

Before we proceed to analyse the results, it is important to define the order parameter. The order parameter $\left\langle \phi_{v}\right\rangle$ represents the average alignment of velocities of agents in a system. It provides insights into the collective order or alignment in the system. Traditionally, it has been studied for phase transition in Vicsek-like models. Mathematically, it is expressed as:


(3.6) $$\begin{array}{ccc} & \langle \phi_{v}\rangle =\frac{1}{N}\left\|\sum_{i=1}^{N}\frac{{\vec{v}}_{i}}{\|{\vec{v}}_{i}\|}\right\| & \end{array}$$

We also define a very useful quantity, radial distribution function g(r), given by:


(3.7) $$\begin{array}{ccc} & g(r)=\frac{1}{\rho N}\sum_{i\neq j}\langle \delta (r-|r_{i}-r_{j}|)\rangle , & \end{array}$$

where the average is performed over the shell volume. g(r) gives a quantitative estimate of the density of particles at distance r from an agent. From a practical point of view, one divides the volume into shells of width Δr at a certain radial distance r and counts the number of agents in that shell [43]. Peaks in g(r) are the signature of different kinds of structure formation in the flock due to stronger attractive forces. Similarly, the troughs in g(r) indicate repulsive forces in the flock.

## 4. Numerical results

We simulate N=100 agents for two-dimensional and N=1000 for the three-dimensional periodic box for $10^{5}$ time steps such that the number density ρ=1 for both two- and three-dimensional. The distance between two agents is the usual Euclidean distance in a periodic box. The speed of agents $v_{0}$ is fixed at 0.5. We choose $|r_{max}-r_{min}|=1$ with two representative r values, $r_{min}=0$ and $r_{min}=1$. $r_{min}=0$ represents the so-called local model, and $r_{min}=1$ is the non-local model. This is because, for $r_{min}>0$, the nearby local agents do not play a role explicitly. We average different features over 10 samples of simulation for each combination of $r_{min}$ and η to obtain statistical stability. As briefly discussed in the introduction section, the experiment on the starling flock favours the number of effective neighbours as low as seven [4]. Thus, the goal of our discussion will be to see how this model performs with as little as one nearest neighbour. We will also contrast the results with the seven nearest neighbours model. The computation was performed using CuPy: a NumPy-Compatible Library for NVIDIA GPU calculations [44] on a workstation with an Intel Xeon W-3265 CPU and A4000 NVIDIA GPU.

**Figure 3.**
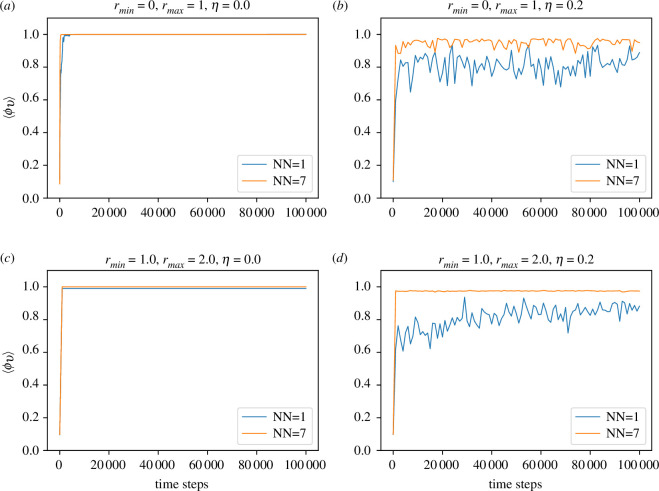
Time evolution of order parameter for the two-dimensional case. NN denotes the number of effective neighbours being considered. It is to be noted that when $\langle \phi_{v}\rangle \approx 1.0$, the lines for NN=1 get buried below that for NN=7. (*a*) for $r_{min}=0.0$ and noise strength η=0.0, (*b*) for $r_{min}=0.0$ and noise strength η=0.2, (*c*) for $r_{min}=1.0$ and noise strength η=0.0 and (*d*) for $r_{min}=1.0$ and noise strength η=0.2.

**Figure 4.**
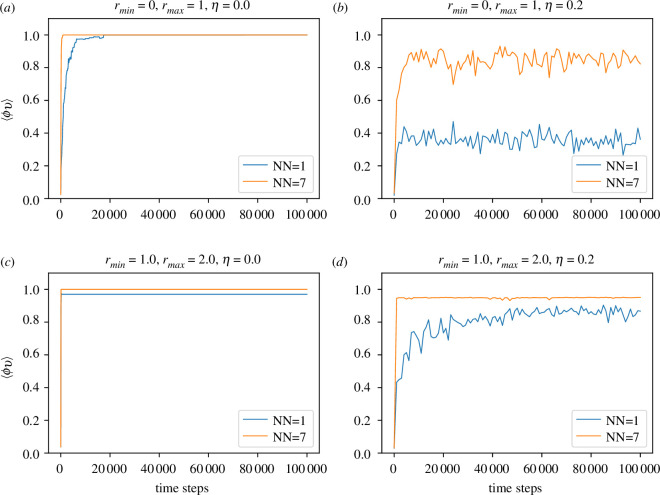
Time evolution of order parameter for the three-dimensional case. NN denotes the number of effective neighbours being considered. It is to be noted that when $\langle \phi_{v}\rangle \approx 1.0$, the lines for NN=1 get buried below that for NN=7. (*a*) for $r_{min}=0.0$ and noise strength η=0.0, (*b*) for $r_{min}=0.0$ and noise strength η=0.2, (*c*) for $r_{min}=1.0$ and noise strength η=0.0 and (*d*) for $r_{min}=1.0$ and noise strength η=0.2.

In figures 3 and 4, we present the evolution of the order parameter $\left\langle \phi_{v}\right\rangle$ with time in two- and three-dimensional cases, respectively, for zero and non-zero noise strength (η=0.2). It is interesting to observe that in the absence of noise (η=0.0), $\left\langle \phi_{v}\right\rangle$ approaches complete order ($\langle \phi_{v}\rangle =1.0$) very quickly even with only one effective nearest neighbour. This is true whether $r_{min}=0$ or $r_{min}=1$ in both two- and three-dimensional cases. Thus, Ricci curvature-induced local interaction is very efficient and economical in generating coherence at a large scale. Also, as expected, with the introduction of noise (η=0.2), the order disrupts, and NN=1 becomes less effective compared to the NN=7 case.

It is to be noted that there is a difference between two- and three-dimensional cases. Unlike the two-dimensional case in figure 3*b*, the three-dimensional case with η=0.2 shown in figure 4*b* says that for $r_{min}=0$, only one effective agent is insufficient for attaining appreciable order. However, this is not true for the non-local case with $r_{min}=1$ as shown in figure 4*d*. With the introduction of not-so-large noise strength (η=0.2), the disruption of order happens for both small and large NN. Yet, the non-local model ($r_{min}=1$) performs much better compared to the local model ($r_{min}=0$).

**Figure 5.**
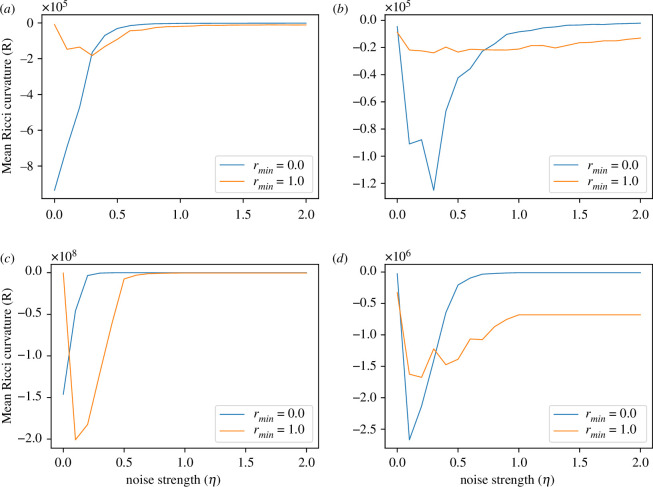
The variation of the mean of the Ricci curvature R as the noise strength (η) varies. (*a*) NN=1 in two dimensions, (*b*) NN=7 in two dimensions, (*c*) NN=1 in three dimensions and (*d*) NN=7 in three dimensions.

Next, we study the mean Ricci curvature for NN=1 and NN=7 in two- and three-dimensional periodic boxes. Here, we have performed the average over all time steps and all agents. In figure 5*a,b*, we present the mean FRC of the vertices with the variation of noise strength η in two dimensions for NN=1 and NN=7, respectively. The three-dimensional counterpart is shown in figure 5*c,d*. We observe that at η=0.0, NN=1 has large negative R for $r_{min}=0$ compared to $r_{min}=1$ (see figure 5*a*,*c*). A cross-over exists between the cases for $r_{min}=0$ and $r_{min}=1$ at non-zero η. For the three-dimensional case, the crossover occurs at a smaller η than the two-dimensional case. Interestingly, the NN=7 cases have a slightly different pattern (see figure 5*b* and *d*). At η=0.0, R for $r_{min}=1$ is more negative compared to $r_{min}=0$. For larger η, $r_{min}=1$ scenarios have lesser R both in two and three dimensions.

The negative R is the signature of the large clustering of agents and edge connectivity. This also tells us about a large information exchange between edges and vertices. A large negative value of R for $r_{min}=0$ at a lower η for NN=1 and subsequent crossover with the $r_{min}=1$ scenario indicates the order in the system is very much sensitive to the strength of the noise. Although η≈0 favours the local model $r_{min}=0$ compared to the non-local $r_{min}=1$ one, as η increases, the local model quickly becomes ineffective. In the three-dimensional case with NN=1, the crossover occurs at a lower η than the two-dimensional case. The point of crossover is pushed to a larger η for NN=7. This is expected because a larger number of neighbours will decide the correct alignment even in the presence of stronger noise.

**Figure 6.**
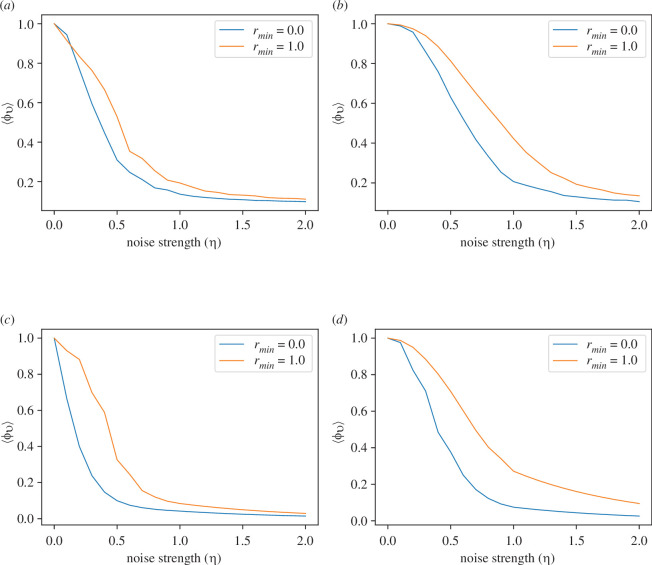
The variation of the mean order parameter $\left\langle \phi_{v}\right\rangle$ as the noise strength (η) varies. (*a*) NN=1 in two dimensions, (*b*) NN=7 in two dimensions, (*c*) NN=1 in three dimensions and (*d*) NN=7 in three dimensions.

In figure 6, we present the often discussed second-order phase transition of the Vicsek models for the two- and three-dimensional periodic boxes. Irrespective of the number of effective neighbours, the phase transition for the non-local model ($r_{min}=1$) takes place at a slightly larger noise strength η compared to the local model ($r_{min}=0$). Also, for NN=7, the system remains aligned in the presence of larger η compared to the NN=1 cases. These observations corroborate the previous discussion on mean Ricci curvature that the non-local models are preferred in the presence of larger noise strength.

**Figure 7.**
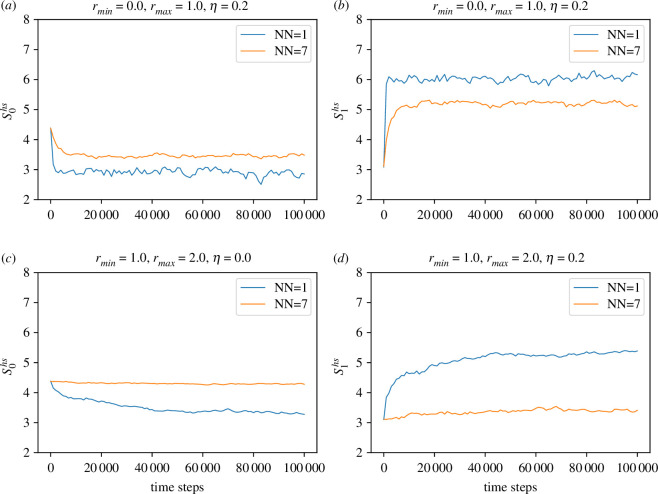
Time evolution of Hodge Spectral entropy $S_{k}^{hs}$ for the local and non-local model in two-dimensional periodic box. (*a*) $S_{0}^{hs}$ with $r_{min}=0$, (*b*) $S_{1}^{hs}$ with $r_{min}=0$, (*c*) $S_{0}^{hs}$ with $r_{min}=1.0$ and (*d*) $S_{1}^{hs}$ with $r_{min}=1.0$.

**Figure 8.**
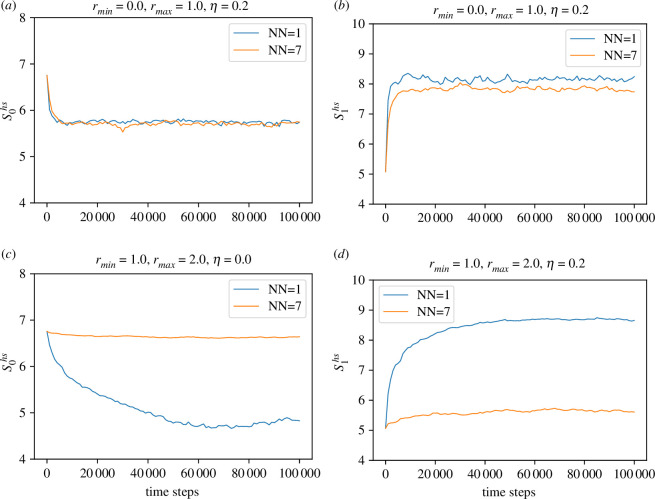
Time evolution of Hodge Spectral entropy $S_{k}^{hs}$ for the local and non-local model in three-dimensional periodic box. (*a*) $S_{0}^{hs}$ with $r_{min}=0$, (*b*) $S_{1}^{hs}$ with $r_{min}=0$, (*c*) $S_{0}^{hs}$ with $r_{min}=1.0$ and (*d*) $S_{1}^{hs}$ with $r_{min}=1.0$.

In figure 7, we present the Hodge spectral entropy ($S_{k}^{hs}$) for the edges and the vertices in two dimensions as a function of time. The *k*-th Hodge spectral entropy measures information transmission across *k*-simplices. In figure 7*a* and *c*, we observe that $S_{0}^{hs}$ approaches a constant value for NN=7. It is to be noted that the zeroth Betti number $\beta_{0}$ is the number of connected components in the simplicial complex. As the number of effective neighbours increases, the system quickly attains appreciable order, even in the presence of noise. Large $S_{0}^{hs}$ indicates large agent-to-agent information flow through the edges connecting each other. We further note that the time evolution of $S_{0}^{hs}$ is more stable for the non-local case than the local one. In the case of a three-dimensional periodic box (see figure 8), the time evolution of $S_{0}^{hs}$ remains similar to that of the two-dimensional periodic box.

$S_{1}^{hs}$ encodes the edge-to-edge information flow. In figure 7*b*,*d*, we observe that $S_{1}^{hs}$ for NN=1 is higher compared to the NN=7 case. However, the difference is higher for the non-local case, indicating a larger difference in the information flow through the vertices in the non-local case. As mentioned earlier, the discussion for Hodge spectral entropies $S_{1}^{hs}$ and $S_{0}^{hs}$ is very much similar for the three-dimensional periodic box (see figure 8). In the three dimensional case, the difference between $S_{1}^{hs}$ for NN=1 and NN=7 is stronger compared to the two-dimensional case. We also note that $S_{k}^{hs}$ remains almost constant with time, indicating continuous information flow for all orders of homology.

**Figure 9.**
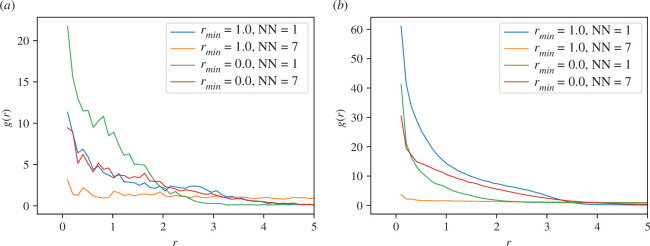
Radial distribution function g(r) with η=0.2. (*a*) in two dimensions and (*b*) in three dimensions.

In figure 9, we present the radial distribution function g(r) for both two- and three-dimensional. In figure 9*a*, we see that for r≈0.0 and NN=1, the local model features larger g(r) compared to the non-local one. The trend is similar for NN=7. Interestingly, the non-local model with NN=1 features the largest g(r) in the three-dimensional case compared to the local one. However, the trend is the opposite for the NN=1 case. Generically, g(r) is almost featureless, as we do not observe any prominent peaks or troughs.

## 5. Conclusion

The study delves into the Ricci curvature-induced dynamics of self-propelled agents within two- and three-dimensional periodic boxes, contrasting local and non-local interaction models. We find that the system can achieve significant order even with a single effective nearest neighbour. Although such a framework is sensitive to noise, the non-local interaction, particularly in the three-dimensional periodic box, is an effective mechanism to create alignment among the agents. Mean Ricci curvature analysis unveils the system’s sensitivity to noise levels, with local models demonstrating better order at lower noise strengths. However, the non-local model features lower mean Ricci curvature for larger noise than the local model. Furthermore, our analysis of Hodge spectral entropy and mean Ricci curvature provides deeper insights into the topological information transmission within the system. Although the radial distribution function is almost featureless, the analysis highlights a nuanced difference between local and non-local interaction models, particularly in the close vicinity of an agent.

Overall, the study underscores the intricate interplay between the nature of the topological interactions, noise strength and dimensionality. The non-local Ricci curvature-induced topological interaction-based model is effective and economical for collective motion in biological systems. These findings contribute to our understanding of complex systems and can help design effective control strategies for collective motion in various real-world applications.

## Data Availability

Data and relevant code for this research work are stored in GitHub: [45] and have been archived within the Zenodo repository: [46].
